# In-vivo dose determination in a human after radon exposure: proof of principle

**DOI:** 10.1007/s00411-022-00972-8

**Published:** 2022-04-04

**Authors:** Franziska Papenfuß, Andreas Maier, Claudia Fournier, Gerhard Kraft, Thomas Friedrich

**Affiliations:** GSI Helmholtzzentrum für Schwerionenforschung GmbH, Planckstraße 1, 64291 Darmstadt, Germany

**Keywords:** Anti-inflammatory therapy, Radon dosimetry, Radiation risk, Occupational and environmental exposure

## Abstract

Radon-222 is pervasive in our environment and the second leading cause of lung cancer induction after smoking while it is simultaneously used to mediate anti-inflammatory effects. During exposure, radon gas distributes inhomogeneously in the body, making a spatially resolved dose quantification necessary to link physical exposure conditions with accompanying risks and beneficial effects. Current dose predictions rely on biokinetic models based on scarce input data from animal experiments and indirect exhalation measurements of a limited number of humans, which shows the need for further experimental verification. We present direct measurements of radon decay in the abdomen and thorax after inhalation as proof of principle in one patient. At both sites, most of the incorporated radon is removed within ~ 3 h, whereas a smaller fraction is retained longer and accounts for most of the deposited energy. The obtained absorbed dose values were $1.5 \pm 0.3$ µGy (abdomen, radon gas) and $1.1 \pm 0.4$ µGy (thorax, radon and progeny) for a one-hour reference exposure at a radon activity concentration of 55 kBq m^−3^. The accumulation of long-retained radon in the abdomen leads to higher dose values at that site than in the thorax. Contrasting prior work, our measurements are performed directly at specific body sites, i.e. thorax and abdomen, which allows for direct spatial distinction of radon kinetics in the body. They show more incorporated and retained radon than current approaches predict, suggesting higher doses. Although obtained only from one person, our data may thus represent a challenge for the barely experimentally benchmarked biokinetic dose assessment model.

## Introduction

Exposure to the radioactive noble gas ^222^Rn is the second leading cause of lung cancer induction after smoking (Darby et al. 2005; Peto and Darby 1994; BEIR 1988) and hence a matter for radiation protection worldwide. As part of the natural decay series of ^238^U, it constantly emanates from rocks and soil (Porstendörfer 1994), so radon gas and its progeny are pervasive in our environment and a major contributor to the annual dose that we receive from ionising radiation (UNSCEAR 2000, 2006). On the other hand, ^222^Rn has been used since medieval times to treat inflammatory diseases such as rheumatoid arthritis or ankylosing spondylitis (Becker 2004). Patients report pain reduction and higher mobility that lasts for 6–12 months after therapy (Dischereit et al. 2014; Franke and Franke 2013). Nevertheless, the underlying mechanism of action is not understood, although there are indications for triggering of the immune system leading to an anti-inflammatory response (Rühle et al. 2017; Kullmann et al. 2019; Shehata et al. 2006) despite typically very low doses. This also raises the question of whether the irradiation of single cells might be responsible for the observed effects or whether nontargeted effects may play a major role in the radiation action of radon (Kullmann et al. 2019; Morgan and Sowa 2005; Kadhim et al. 2013).

The main route of intake for radon and its progeny is inhalation (Smerajec and Vaupotič 2012; Maier et al. 2021; ICRP 2017; UNSCEAR 2017). Considering radon gas, simulations predict that under steady-state-conditions only approximately 1% of the inhaled radon is absorbed into the blood and distributed all over the body, where it dissolves and accumulates in fat-rich organs (Sakoda et al. 2010). Radon decay in air leads to highly charged progeny, which become neutralised rapidly by interaction with trace gases and can attach to aerosols of different sizes which leads to a certain activity size distribution in the ambient air (Chu and Hopke 1988; Hopke 1996). During inhalation, the progeny associated with the unattached and attached activity size distribution is deposited in the respiratory tract by different mechanisms that are mainly dependent on particle size. They accumulate with every breath (Carvalho et al. 2011) and decay so that they deposit their energy there as nearly no efficient clearance occurs (ICRP 1994; Stuart 1984). The low transfer of radon into the blood during inhalation and the efficient deposition of inhaled decay products in the respiratory tract is at least one of the reasons why radon progeny is predicted to account for more than 95% of the effective dose. This is also promoted by the comparably low target cell distance of the progeny in the respiratory tract. The lung, where progeny are absorbed, is believed to contribute over 95% to the effective dose (ICRP 2017).

Incorporated ^222^Rn decays via the α-emitting isotopes ^218^Po and ^214^Po and the β- and γ-emitting isotopes ^214^Pb and ^214^Bi to ^210^Pb (see decay scheme below) (Sóti et al. 2019). The α-emitting isotopes contribute in total with 19.18 MeV to most of the deposited energy, while β- and γ-radiation, which make up for approximately 10% of the decay energy, can escape the body, which leads to negligible small dose contributions of these components. Due to the long half-life of ^210^Pb of 22.3 years, further decay products become biologically irrelevant.

$$\begin{array}{*{20}c} {{}^{222}{\text{Rn}}} \\ {{3}{\text{.8 d}}} \\ \end{array} \;_{\overrightarrow{\alpha\; { 5}{\text{.49 MeV}}}}\; \begin{array}{*{20}c} {{}^{218}{\text{Po}}} \\ {{3}{\text{.05 min}}} \\ \end{array}  \; _{\overrightarrow{{\alpha\; { 6}{\text{.00 MeV}}}^{^{}}}}\;\begin{array}{*{20}c} {{}^{214}{\text{Pb}}} \\ {{26}{\text{.8 min}}} \\ \end{array} \;_{\overrightarrow{\beta , \gamma\; }}^{}\begin{array}{*{20}c} {{}^{214}{\text{Bi}}} \\ { 19.9 \min } \\ \end{array} \;_{\overrightarrow{\beta , \gamma }}^{}\begin{array}{*{20}c} {{}^{214}{\text{Po}}} \\ {{ 164 }\mu {\text{s}}} \\ \end{array} \;_{\overrightarrow{\alpha\; { 7}{\text{.69 MeV}}}^{}} \begin{array}{*{20}c} {{}^{210}{\text{Pb}}} \\ {{22}{\text{.3 a}}} \\ \end{array} .$$

For both, proper risk assessment and investigations concerning potential anti-inflammatory effects, spatially and temporally resolved dose determination is essential. Currently used dose assessment models by the International Commission on Radiological Protection (ICRP) are based on two different concepts. In epidemiological studies the lung cancer risk arising from chronical internal densely ionizing radiation due to exposure to certain radon activity concentrations and the total detriment in the A-bomb survivors after short-term exposure to a certain effective dose of sparsely ionizing radiation are determined. By comparing the risk values, the effective dose for radon exposure is derived. Therefore, two differing exposure scenarios are compared and there are uncertainties concerning the approximation of the effective dose or radon activity concentration the people were exposed to. Moreover, no predictions concerning the spatial and temporal radon distribution in the body can be made (UNSCEAR 2017; Müller et al. 2016; ICRP 2015; Little 1997). In the dosimetric approach, the lung equivalent dose is estimated by simulation of the distribution of radon decay products in the respiratory tract taking into account its morphology and physiology in combination with the physical properties of the inhaled particles (UNSCEAR 2017; Müller et al. 2016; ICRP 2015; Little 1997). To estimate the equivalent doses to other organs, a biokinetic model is used to simulate the radon distribution in the body after inhalation (ICRP 2017). Here, uncertainties arise from the used input parameters. In the model, the body is divided into compartments with specific physiological properties based on the anatomic and physiological properties of the ICRP “reference man” (Leggett et al. 2013; ICRP 1975). Then, so-called partition coefficients, serve as input parameters. They are solely gained from one animal experiment in rats by Nussbaum and Hursh (Nussbaum and Hursh 1957), who measured radon solubility given by the ratio of the radon concentration per ml of the investigated tissue and air. The solubility ratio of two compartments gives the corresponding partition coefficient. The so obtained data are directly used in the models or extrapolated to different body compartments, for which no experimental data are available (Khursheed 2000; NRC 1999). This gives rise to the issue of transferability of data from one organism to the other because of anatomical and physiological differences. The so-developed biokinetic model (Leggett et al. 2013) was benchmarked by measurements of the change in radon activity in exhaled air of only two individuals following radon inhalation (Harley et al. 1994). Additionally, a total-body retention of radon after ingestion is reported by three authors with five individuals in total (Andersson and Nilsson 1964; Gosink et al. 1990; Hursh et al. 1965) that showed different radon kinetics, so that the transfer time of radon from the stomach to the small intestine had to be adapted to properly describe the obtained data (ICRP 2017).

In general, epidemiological studies do not allow us to determine the spatial and temporal radon distribution in the body. Biokinetic models are able to do so, but the proposed kinetics and derived dose values are verified only by a limited number of heterogeneous data sets. In addition to our work presented here, there are only two further measurements for radon inhalation in humans by Harley (Harley et al. 1994) and a more recent publication by Fojtik (Fojtik et al. 2020) with only the first to be considered by the ICRP. All in all, both concepts of dose assessment models would profit from enlargement of experimental data and measurements in human.

**Fig. 1:**
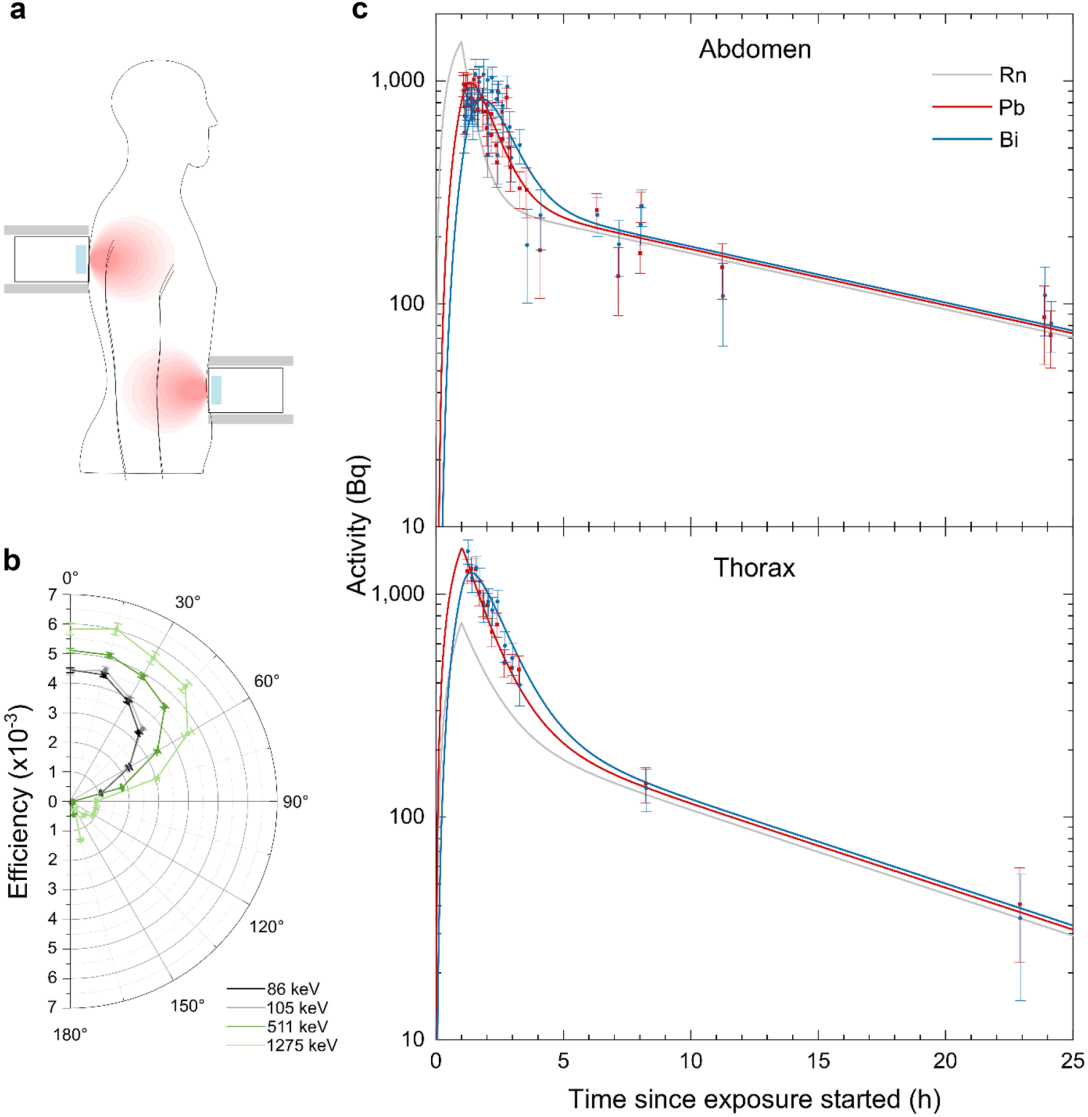
**a** Recording of γ-spectra at the abdominal and thoracic body sites. The Ge crystal is illustrated in blue, and a lead shielding for background reduction is illustrated in grey. The intensity of the red areas reflects the detector efficiency. **b** Measurement of detector efficiency for different azimuthal angles at a distance of 0.5 m from the middle of the detector cap. The angle 0° represents the measurement at the detector front and 180° at the back. **c** Scaled (55 kBq m^−3^, 40.32 kg) and pooled activity values for ^214^Pb (red) and ^214^Bi (blue) as well as fitted activity curves for ^214^Pb, ^214^Bi and ^222^Rn (grey) in the abdomen and thorax. Data points are optically shifted (− 1 min for ^214^Pb and + 1 min for ^214^Bi) for better data representation (*n* = 3 abdomen, *n* = 1 thorax)

We present direct activity measurements of decaying ^214^Pb and ^214^Bi in the abdomen and thorax of a human starting only a few minutes after radon inhalation (Fig. 1a, b). The time course of the activity of these isotopes was followed for up to 24 h, allowing conclusions on the change in radon activities in the respective measured site. Accordingly, the deposited energy and thus locally absorbed doses were determined without the need for any model assumptions. Notably, radon is detected at the site where it decays, allowing direct correlation and location of the origin of long-retained radon in the body. As a result, we observed varying radon kinetics at the different measured sites and determined the absorbed and equivalent dose from incorporated radon gas to the abdomen and thorax. Our measurements show higher radon activities in the body and thus higher doses from incorporated radon gas than predictions by current radiation protection models suggest. This raises the question of the role of primary radon in the assessment of the risk related to radon exposure, as this component is generally neglected due to its small anticipated contribution to the effective dose compared to the lung equivalent dose from incorporated progeny. Additionally, the role of the hit probability of cells during radon therapy is discussed with respect to the proposed underlying mechanism of potential medical action.

## Methods

### Exposure

A voluntary patient was exposed in a radon inhalation gallery (Acuradon, Bad Kreuznach, Germany) for one hour at radon activity concentrations ranging from 27 to 77 kBq m^−3^. During the time of exposure, the radon activity concentration was nearly constant (fluctuations were less than 7%) but varied between different days. It was monitored by the gallery holder with the AlphaGUARD device (Bertin Instruments, Montigny-le-Bretonneux, France). During exposure, the ambient air was actively filtered, resulting in a low progeny concentration (*F* factor $< 0.1$) and a relatively high amount of unattached progeny ($f_{{{\text{unatt}}}} > 70 \%$). The progeny concentration in air and the fraction of unattached and attached nuclides were measured during every exposure using the AZ-3 measurement device (α-Kernstrahlungsmesstechnik, Chemnitz, Germany). After exposure, the voluntary patient changed clothes and was scanned with the LB 124 SCINT device (Berthold Technologies GmbH & Co. KG, Bad Wildbad, Germany) to exclude surface contamination that could influence the detected signal.

### Recording of γ-spectra

We used γ-spectroscopy to determine the activity curves of the γ-emitting radon decay products ^214^Pb and ^214^Bi. For recording, we used a nitrogen-cooled, portable semiconductor detector (BE3825 Mirion Technologies GmbH, Rüsselsheim, Germany) with a high purity Ge crystal. Throughout every measurement, the detector was adjusted in a specific position in the room for abdominal and thoracic measurement to allow reproducibility. Additionally, the position of the volunteer in front of the detector was marked on a shirt worn for the measurements. For recordings at the abdominal site, the detector was positioned directly on the stomach 2–3 cm over the navel and for the thoracic site on the right shoulder blade to have the lung directly in the detector field. The measurements for the short-term component took place in an infrastructure room outside the radon gallery, whereby measurements of later time points were mainly conducted in a room at the GSI Helmholtzzentrum für Schwerionenforschung GmbH.

Before every exposure in the radon gallery, a background spectrum of the volunteer was recorded. After exposure and changing of clothes, the volunteer was positioned in front of the detector, and γ-spectra were recorded. The recording time was adapted to the present activities. The measurements started not less than 5 min after exposure, and depending on the activity, recording times for one spectrum were at least 5 min. During recording, the volunteer usually had a light meal and had short breaks to move before the next measurement started. After the first ~ 3 h, there was a break for approximately 1.5 h before starting the next measurement session, in which γ-spectra were recorded in varying intervals for up to 24 h, whereby the recording time could be up to 1 h. In total, four measurements took place at the abdominal site and one measurement was conducted at the thoracic site.

During the recording of the γ-spectra, we monitored the radon activity concentration in the measurement room using the AlphaGUARD P30 (Bertin Instruments, Montigny-le-Bretonneux, France). Moreover, γ-spectra with a filled water container (38 cm long, 20.25 cm deep and 24.5 cm high) were recorded in between measurements of the volunteer for further background analysis.

### Activity calculation

To calculate the activity $A$ of ^214^Pb and ^214^Bi from the spectra, we used the following Eq. 1:

1 $$A = \frac{{N_{{\text{n}}} \cdot f_{1} \cdot f_{2} }}{\varepsilon \cdot p \cdot T}.$$

There, $N_{{\text{n}}}$ is given by the background adjusted net peak area underneath a specific peak in the spectrum, $f_{1}$ is the decay correction, $f_{2}$ the pile-up correction, ε the detector efficiency, $p$ is the branching ratio for the detected emission lines (Chu et al. 1999) and $T$ the recording time of one spectrum. For the calculation of $N_{{\text{n}}}$, the ^214^Pb peak at an emission energy of 352 keV and sometimes 295 keV, just as the ^214^Bi peak at 609 keV, were evaluated in the recorded spectra after exposure and the background measurements using Genie 2000 peak analyses software (Mirion Technologies GmbH, Rüsselsheim, Germany). Due to logistical reasons, the measurement time for the background spectra of the volunteer in Bad Kreuznach was too short to evaluate the ^214^Pb peaks. Therefore, we recorded background spectra with the aforementioned water container as shielding. The volunteer background at our institute and in Bad Kreuznach was then calculated by comparing the recorded ^214^Bi activities. From the ^214^Bi-line transformation factors for the background at GSI to Bad Kreuznach and for the difference in the effective absorber length of the water container and the volunteer were determined. For this procedure, a constant background in both places had to be assumed, which was validated by background measurements at different days and daytimes.

The decay correction $f_{1}$ is determined with an iterative procedure by fitting an activity model to the obtained nondecay-corrected activity values for ^214^Pb and ^214^Bi. Then, the decay correction was determined by

2 $$f_{1} = \frac{{T \cdot A\left( {t_{i} } \right)}}{{\mathop \smallint \nolimits_{{t_{i} }}^{{t_{i} + T}} A\left( t \right){\text{d}}t}},$$

where $t_{i}$ is the time at which the recording started,$A\left( {t_{i} } \right)$ the measured activity at that time and $T$ is the recording time. After determination of $f_{1}$ for every single activity value, the decay-corrected values are calculated and the procedure is repeated. After three iterations, the obtained activity values change only marginally.

The pile-up correction $f_{2}$ and the detector efficiency *ε* are determined using a detector-adapted so-called geometry editor software (Mirion Technologies GmbH, Rüsselsheim, Germany). This software calculates both values for editable simple geometries. Cross-check measurements with calibration sources agree fairly well with the obtained efficiency values, although they are systematically slightly underestimated (~ 10%). For the abdominal geometry, a centred cuboid (28 cm long, 24 cm deep and 60 cm high) with a density of water is assumed. For the thoracic site, the same geometry is used, but with the detector positioned at the upper edge instead centred at the cube. The pile-up correction for both geometries is negligibly small ($f_{2} = 1$). For clarity, in Table 1 an overview of all denotations used in the equations throughout this work is given.

**Table 1:** Overview of quantities used in the equations

Quantity	Explanation
$A$	Activity
$a$	Conversion factor for calculation of potential α-energy concentration at a given equilibrium equivalent concentration
$A_{{{\text{inc}}}}$	Incorporated activity
$c_{{{\text{Rn}}}}$	Radon activity concentration in ambient air
$D$	Absorbed dose
$E_{{{\text{ip}}}}$	Energy deposition by directly incorporated progeny
$\varepsilon$	Detector efficiency
$\tilde{F}$	Equilibrium factor
$F_{1}$	Blood perfusion rate compartment 1
$F_{2}$	Blood perfusion rate compartment 2
$f_{1}$	Decay correction
$f_{2}$	Pile-up correction
$f_{{{\text{unatt}}}}$	Fraction of unattached progeny
$k_{j}$	Detected number of absorbed nuclide *j* in the respiratory tract at a given radon activity concentration
$k_{{{\text{Rn}}}}$	Ratio of radon activity concentration in air and arterial blood
$\lambda_{j}$	Decay constant of nuclide *j*
$\lambda_{{\text{RT - env}}}$	Transition rate of radon from respiratory tract to environment
$m$	Mass of measured site
$N_{j}$	Number of atoms of nuclide *j* in measured site
$N_{n}$	Net peak area
$p$	Branching ratio
$P_{1}$	Partition coefficient compartment 1
$P_{2}$	Partition coefficient compartment 2
${\text{RMV}}$	Respiratory minute volume
$T$	Recording time
$t$	Time
$t_{\exp }$	Exposure time
$t_{i}$	Start time of recording
$V_{1}$	Volume compartment 1
$V_{2}$	Volume compartment 2
${\text{VRT}}$	Respiratory tract volume

### Simulation of activity curves

The activity measurements show that both measured sites, the abdomen and thorax, have to be divided into at least two compartments. In the abdominal site, there is no directly incorporated progeny, as transport rates of inhaled progeny are too slow to efficiently reach the detected site. Moreover, we can neglect the transport processes of radon decay products in the body, as they show lower rates than the decay constant of the nuclides (ICRP 1994). Therefore, the detected signal is caused only by incorporated radon that decays in the measured site and its progeny. Inspired by the approach by Leggett et al. (Leggett et al. 2013), the transport of radon in and out of the measured site is given by

3 $$\begin{gathered} \frac{{{\text{d}}N_{{{\text{Rn}},{\text{in}}}} }}{{{\text{d}}t}} = \frac{{F_{1} }}{{k_{{{\text{Rn}}}} \lambda_{{{\text{Rn}}}} }}c_{{{\text{Rn}}}} + \frac{{F_{2} }}{{k_{{{\text{Rn}}}} \lambda_{{{\text{Rn}}}} }}c_{{{\text{Rn}}}} , \hfill \\ \frac{{{\text{d}}N_{{{\text{Rn}},{\text{out}}}} }}{{{\text{d}}t}} = \frac{{F_{1} }}{{V_{1} P_{1} }}N_{{\text{Rn,1}}} + \frac{{F_{2} }}{{V_{2} P_{2} }}N_{{\text{Rn,2}}} . \hfill \\ \end{gathered}$$

The parameter $N_{Rn}$ gives the number of ^222^Rn atoms in the measured site and $c_{{{\text{Rn}}}}$ the radon activity concentration in ambient air. The parameter $\lambda_{{{\text{Rn}}}}$ represents the physical decay constant of radon. The numbers 1 and 2 represent the two compartments within the measured site and $F$ their blood perfusion rates, $V$ their volumes and $P$ their partition coefficients. The constant $k_{{{\text{Rn}}}}$ specifies the ratio of the radon activity concentration in air and arterial blood and is dependent on individual physiological parameters and the physical activity during exposure. Combining these terms and accounting for the radioactive decay of radon, we obtain the temporal change of ^222^Rn

4 $$\frac{{{\text{d}}N_{{{\text{Rn}}}} }}{{{\text{d}}t}} = \frac{{F_{1} }}{{k_{{{\text{Rn}}}} \lambda_{{{\text{Rn}}}} }}c_{{{\text{Rn}}}} + \frac{{F_{2} }}{{k_{{{\text{Rn}}}} \lambda_{{{\text{Rn}}}} }}c_{{{\text{Rn}}}} - \frac{{F_{1} }}{{V_{1} P_{1} }}N_{{\text{Rn,1}}} - \frac{{F_{2} }}{{V_{2} P_{2} }}N_{{\text{Rn,2}}} - \lambda_{{{\text{Rn}}}} N_{{{\text{Rn}}}} .$$

Here, $\frac{{F_{{\text{i}}} }}{{k_{{{\text{Rn}}}} \lambda_{{{\text{Rn}}}} }}$ represents the transport coefficient of the radon activity concentration into the compartment $i$ and $\frac{{F_{{\text{i}}} }}{{V_{{\text{i}}} P_{{\text{i}}} }}$ represents the removal coefficient of radon out of the two compartments. Because of ^222^Rn decay, ^218^Po is built, which decays to ^214^Pb and then to ^214^Bi, the differential equation for the temporal change of these atoms in the measured site is given by

5 $$\begin{gathered} \frac{{{\text{d}}N_{{{\text{Po}}}} }}{{{\text{d}}t}} = \lambda_{{{\text{Rn}}}} N_{{{\text{Rn}}}} - \lambda_{{{\text{Po}}}} N_{{{\text{Po}}}} , \hfill \\ \frac{{{\text{d}}N_{{{\text{Pb}}}} }}{{{\text{d}}t}} = \lambda_{{{\text{Po}}}} N_{{{\text{Po}}}} - \lambda_{{{\text{Pb}}}} N_{{{\text{Pb}}}} , \hfill \\ \frac{{{\text{d}}N_{{{\text{Bi}}}} }}{{{\text{d}}t}} = \lambda_{{{\text{Pb}}}} N_{{{\text{Pb}}}} - \lambda_{{{\text{Bi}}}} N_{{{\text{Bi}}}} . \hfill \\ \end{gathered}$$

The parameter $\lambda_{j}$ represents the decay constant and $N_{j}$ the number of the corresponding nuclide $j$. Initial conditions for solving the set of differential equations are that there is no radon and progeny at the beginning of exposure and $c_{{{\text{Rn}}}}$ becomes approximately 0 after the 1-h exposure, so that there is no transport of radon into the measured site anymore. Solving the system of linear differential equations gives the temporal change in nuclides in the measured site. By multiplying the solution by the decay constant of the corresponding nuclide, the activity curves are given in terms of the quantities $\frac{{F_{1} }}{{k_{{{\text{Rn}}}} \lambda_{{{\text{Rn}}}} }}$, ${ }\frac{{F_{2} }}{{k_{{{\text{Rn}}}} \lambda_{{{\text{Rn}}}} }}$, $\frac{{F_{1} }}{{V_{1} P_{1} }}$ and $\frac{{F_{2} }}{{V_{2} P_{2} }}.$ These quantities reflect the strength and steepness of both components and were the four fit parameters that were adapted to obtain the curves that describe the experimentally obtained ^214^Pb and ^214^Bi activities.

In the thoracic region, we additionally have to account for directly incorporated progeny. By doing so, the differential equations for the progeny read

6 $$\begin{gathered} \frac{{{\text{d}}N_{{{\text{Po}}}} }}{{{\text{d}}t}} = k_{{{\text{Po}}}} c_{{{\text{Rn}}}} + \lambda_{{{\text{Rn}}}} N_{{{\text{Rn}}}} - \lambda_{{{\text{Po}}}} N_{{{\text{Po}}}} , \hfill \\ \frac{{{\text{d}}N_{{{\text{Pb}}}} }}{{{\text{d}}t}} = k_{{{\text{Pb}}}} c_{{{\text{Rn}}}} + \lambda_{{{\text{Po}}}} N_{{{\text{Po}}}} - \lambda_{{{\text{Pb}}}} N_{{{\text{Pb}}}} , \hfill \\ \frac{{{\text{d}}N_{{{\text{Bi}}}} }}{{{\text{d}}t}} = k_{{{\text{Bi}}}} c_{{{\text{Rn}}}} + \lambda_{{{\text{Pb}}}} N_{{{\text{Pb}}}} - \lambda_{{{\text{Bi}}}} N_{{{\text{Bi}}}} . \hfill \\ \end{gathered}$$

The constants $k_{{{\text{Po}}}}$, $k_{{{\text{Pb}}}}$ and $k_{{{\text{Bi}}}}$ represent how many nuclides are absorbed in the respiratory tract at a certain radon activity concentration ($c_{{{\text{Rn}}}}$) and are detected during measurement. The constants are dependent on the *F* factor and $f_{{{\text{unatt}}}}$ and thus vary from exposure to exposure. All include the unknown parameter $k$ that represents which fraction of absorbed nuclides is detected during measurement. Further procedure is analogous to that for the abdominal measurements, only with the additional fit parameter $k$.

As all measurements were conducted at different radon activity concentrations during exposure (27–77 kBq m^−3^), the recorded ^214^Pb and ^214^Bi activities were linearly scaled to 55 kBq m^−3^ (a typical radon activity concentration in radon inhalation galleries) for a joint analysis. This way, we received activities as if the activity concentration was that reference. When individual nonscaled data sets were fitted, the number of fit parameters was reduced, as we used the value for the removal coefficient for radon and the ratio of the perfusion rates of the two compartments of the measurement region from the pooled analysis because the tissue composition of each body site remained the same. We fitted the so-developed model to the measured activities of ^214^Pb and ^214^Bi to obtain the values for the free fit parameters. By inserting these into the solution of the differential equations for the nuclides and multiplying it with the corresponding decay constants, the activity curves for ^214^Bi, ^214^Pb, ^218^Po and ^222^Rn are obtained.

### Determination of absorbed doses and dose conversion factors

From the obtained activity curves of radon and its progeny, the absorbed dose can be calculated for both body sites. As no directly incorporated progeny reach the abdominal site, all of the absorbed dose originates solely from incorporated radon gas. Thus, the area underneath the activity curves, which gives the total number of decays, should be the same for all nuclides as transport rates of radon progeny in the body are much lower than their decay constants. Radon and its progeny emit a summed up α-energy of 19.18 MeV until they decayed to ^210^Pb and the contribution of β- and γ-decays to the absorbed dose is small due to their lower emission energies and higher range in tissue. By considering the equivalent doses that take into account the biological effectiveness of α-radiation with a radiation factor of 20 compared to β- and γ-radiation with 1 (ICRP 2003), the contribution of β- and γ-decays becomes even more neglectable. By multiplying the potential α-energy of radon by the number of decaying radon atoms, the deposited energy in the measured site is obtained. By dividing by the mass of the measured site $m$, the absorbed dose $D$ is determined

7 $$D = \frac{{\mathop \smallint \nolimits_{0}^{\infty } A_{{{\text{Rn}}}} \left( t \right){\text{d}}t\cdot19.18 {\text{MeV}}}}{m}.$$

Additionally, the absorbed dose in compartment 1 and 2 can be determined by separating the activities originating from the different compartments. Errors are gained with Gaussian error propagation with respect to the uncertainties of the fit parameters.

In the thoracic site, the absorbed dose due to incorporated radon is calculated in the same way as for the abdominal site, but energy deposition due to directly incorporated progeny $E_{{{\text{ip}}}}$ also plays a role and is determined for each nuclide separately with the following equation:

8 $$E_{{{\text{ip}}}} = t_{{{\text{exp}}}} \cdot {\text{RMV}} \cdot c_{{{\text{Rn}}}} \cdot a \cdot \tilde{F} \cdot \left( {0.984 \cdot f_{{{\text{unatt}}}} + 0.3 \cdot \left( {1 - f_{{{\text{unatt}}}} } \right)} \right).$$

Here, ${\text{RMV}}$ gives the respiratory minute volume which was assumed to be $0.48\frac{{m^{3} }}{h}$ for a resting but not sleeping person on basis of the ICRP (ICRP 1975). The parameter $a$ = $\frac{{1.3 \cdot 10^{ - 8} }}{3750}$ MeV Bq^−1^ is the conversion factor to calculate the potential α-energy concentration at a given equilibrium equivalent concentration, $\tilde{F}$ the *F* factor and $f_{{{\text{unatt}}}}$ the fraction of the unattached progeny that were measured during the exposure of the volunteer. The factors 98.4% and 30% represent the fraction of inhaled progeny associated with the unattached and attached activity size distribution that were absorbed in the respiratory tract during inhalation (ICRP 2017).

To determine the dose conversion factor, the incorporated activity $A_{{{\text{inc}}}}$ for an exposure for a certain time $t_{{{\text{exp}}}}$ at a given radon activity concentration $c_{{{\text{Rn}}}}$ is calculated by

9 $$A_{{{\text{inc}}}} = c_{{{\text{Rn}}}} \lambda_{{\text{RT-env}}} V_{{{\text{RT}}}} t_{\exp } ,$$

with the transition rate of radon from the respiratory tract to the environment $\lambda_{{\text{RT-env}}}$ and the respiratory tract volume $V_{{{\text{RT}}}}$(ICRP 2017). This leads to an incorporated activity of 22.9 kBq for a one-hour exposure at 55 kBq m^−3^. The measured absorbed dose is transferred into an equivalent dose for the measured site using a radiation weighting factor of 20 for α-particles (ICRP 2003). The dose conversion factor is obtained by dividing the equivalent dose by the incorporated activity.

### The adapted simplified model used by the ICRP

For comparison, the currently used model by the ICRP (2017) for dose predictions in radiation protection (Leggett et al. 2013) is simplified and adapted for measurement at the abdominal site of our volunteer. For simplification, we divided the body into five compartments, fat1, fat2, venous blood and arterial blood, which correspond to those in the original model by Leggett and combined all other compartments of that model to one. Moreover, the compartment volumes were adapted to the individual abdominal site of the volunteer. As the volunteer has a similar weight as the ICRP reference man, we adapted the tissue volumes, whereby the fatty tissue is equally attributed to fat compartments 1 and 2 (Leggett et al. 2013). As fat compartment 2 represents poorly perfused fat tissue, we correlated it with the subcutaneous fat content, for which studies show that half of it is located in the trunk (Störchle et al. 2018). Under the assumption that 50% of the whole-body fat content, 70% of the blood and 50% of the other tissue are located in the trunk and normalising this to the 40.32 kg of the presumed abdominal geometry, we obtained the adapted composition of the abdominal site. The activity curves for ^214^Pb and ^214^Bi were simulated for these assumptions and could be directly compared with the experimentally obtained data.

## Results

### Time course of activities and retention times for radon and its progeny at different body sites

The time evolution of the activities of the γ-emitting decay products ^214^Pb and ^214^Bi in the abdomen and thorax of a 77-year-old volunteer (age at first measurement) after a one-hour exposure in an inhalation gallery was measured. As the radon activity concentration varied between different exposures, all recorded activities were scaled to a radon activity concentration of 55 kBq m^−3^ (a typical radon activity concentration in radon inhalation galleries) using a linear relation between the measured activities and the radon activity concentration during exposure, which was confirmed in the next section (see “Methods” section: simulation of activity curves for further details). The result in Fig. 1c shows a nearly exponential decrease in the ^214^Pb activities at both measured sites at early times after exposure, which is due to the decay of the accumulated ^214^Pb during the exposure time and the newly produced ^214^Pb from retained decaying radon. A temporary build-up in the ^214^Bi activities due to the decay of the mother nuclide ^214^Pb is observed. When ^214^Pb is decayed, the ^214^Bi activities also approximate a pure exponential decay. However, we observe higher activities at later times (starting ~ 3 h after exposure) than would be expected from the course of the activities in the first 3 h. Therefore, the retention of radon gas within the body must consist of at least two mechanisms: one in which most of the radon is absorbed while being retained for a short time and a second in which a smaller fraction of the incorporated radon is retained longer. Thus, two functional compartments that reflect the differing retention times of radon were assumed in both measured sites. By fitting an empirical two-component model approach that considers the decay cascade of radon to the measured data (see the Methods section), we obtain the activity curves for ^222^Rn, ^214^Pb and ^214^Bi. From these curves, the retention times of radon in the two compartments at each measured site are extracted as given in Table 2. These data are based on three measurements at the abdominal and one at the thoracic site.

**Table 2:** Biological half-lives of short- and long-retained radon at different body sites

Body site	Abdomen	Thorax
Half-life short-retained radon (min)	20.7 ± 3.1	47.8 ± 30.9
Half-life long-retained radon (h)	13.8 ± 3.8	8.8 ± 3.0

Values were obtained from an empirical model approach with two retention components fitted to the measured activity values of ^214^Pb and ^214^Bi after radon exposure in a voluntary patient. The uncertainties are determined by the error of the fit parameters

The retention times depend on the blood perfusion rate, volume and partition coefficient of each compartment (Leggett et al. 2013). The comparison of the biological half-lives for radon in the abdomen and thorax (Table 2) shows different retention times that can be explained by the physiological properties of the measured site, i.e., varying perfusion rates and radon solubilities in different tissues. From in vitro measurements, it is known that radon solubility is approximately a factor of 60 higher in oleic acid than in isotonic saline solution (Sanjon et al. 2019). Thus, fatty tissue is expected to show higher biological half-lives for radon and seems to be the origin of the measured long-retained radon which is also supported by the literature (NRC 1999). The reduced half-life of long-retained radon in the thorax compared to the abdomen indicates a higher perfusion rate of the fatty tissue at this site or a lower solubility of radon due to a different tissue composition. Additionally, a mixture of the signal from the fatty tissue with another tissue, which has a lower biological half-life and is efficiently detected during the measurement at the thoracic site, would result in a lower retention times in the thorax than in the abdomen.

### Doses and their dependence on the radon activity concentration during exposure

**Fig. 2:**
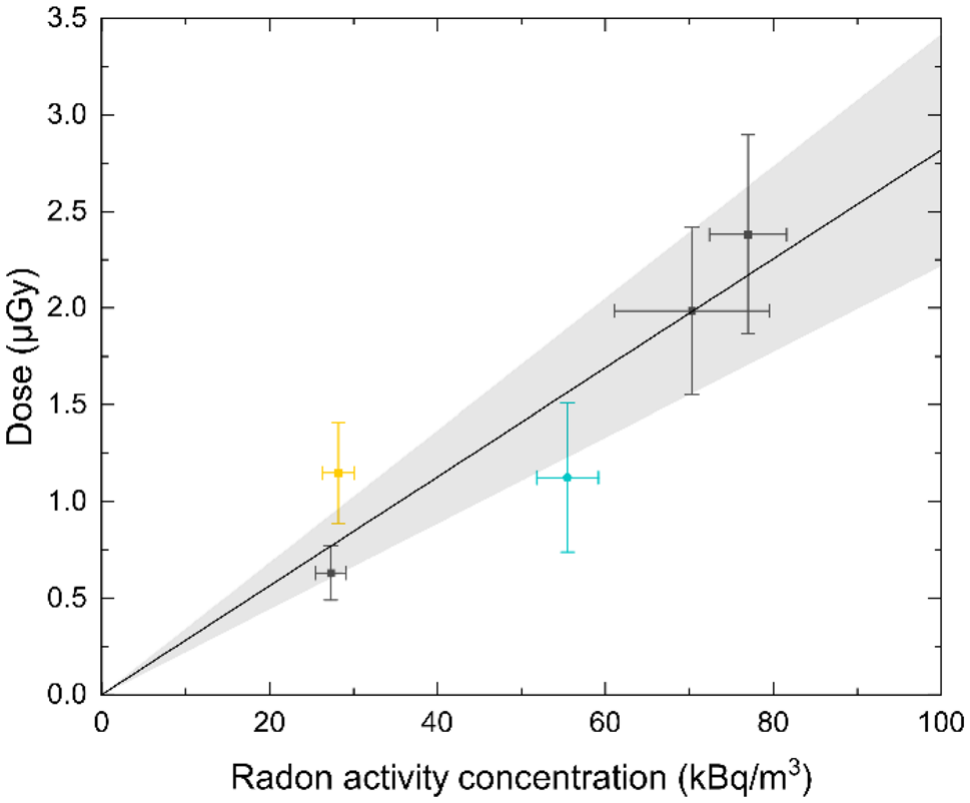
Absorbed dose values for a one-hour exposure at different radon activity concentrations in the abdominal (black) and thoracic body site (blue) as well as for the measurement at the abdominal site at a higher physical activity of the volunteer during exposure (yellow). The black line represents the absorbed dose values and their error margin (shaded area) that are expected from the pooled analysis of the data assuming a linear relation between the absorbed dose and radon activity concentration during exposure. Errors are gained with Gaussian error propagation with respect to the uncertainties of the fit parameters

To calculate the absorbed doses to the measured sites, as depicted in Fig. 2, we evaluated the integral of the time-dependent activity curves, which gives the number of decaying atoms, multiplied this value by the decay energies of radon and its progeny and normalised the calculated deposited energy to the mass of the measured sites. For the abdominal site, the energy is deposited solely by incorporated radon gas and its progeny, whereas in the thoracic site, directly inhaled progeny are also accounted for dose application, whereby the amount of progeny in the ambient air in the gallery is reduced due to active filtration. For the results presented below, homogeneous activity and consequently homogenous energy deposition in the measured sites were assumed. The accompanying limitations of this assumption will be discussed later.

First, all abdominal or thoracic activity measurements were normalised to a reference radon activity concentration of 55 kBq m^−3^ during exposure. By assuming homogeneous energy deposition in the measured site, the absorbed dose values for the pooled analysis for a one-hour reference exposure at 55 kBq m^−3^ are given by $1.5 \pm 0.3$ µGy in the abdomen and by $1.1 \pm 0.4$ µGy in the thorax. In the next step, the determined dose values can be rescaled to the actual radon activity concentrations during the exposures (Fig. 2, black line). Then, the dose values for the nonscaled data sets were calculated (Fig. 2, single data points). They show good agreement with the rescaled values from the pooled analysis, which validates the linear relation between the radon activity concentration during the exposure and the absorbed dose in the volunteer.

In one exception, in which our model did not appropriately fit the measured activity curves, the volunteer’s physical activity during exposure was enhanced compared to other exposure scenarios (conversation instead of rest/sleep), which resulted in a differing kinetic for the radon distribution within the body. Especially the short-term component appeared to be very pronounced so that the yellow data point in Fig. 2 (evaluated by the fit adapted for the resting volunteer) that already indicates an enhanced absorbed dose would be even higher. This suggests a relation between the absorbed dose from radon exposure and both the individual physiological properties of the volunteer and its physical activity during exposure. Additionally, current dose assessment models predict a positive correlation between physical activity and the dose (ICRP 2017; Leggett et al. 2013), although no experiments verifying this dependence have been conducted so far. One may expect that physical activity likewise may enhance therapeutic effects of radon at equal dosage, or alternatively allow to shorten exposure times. However, this can only be investigated following proper clinical studies for established regimen settings as reference.

### Dose from short- and long-retained radon and directly inhaled progeny

**Fig. 3:**
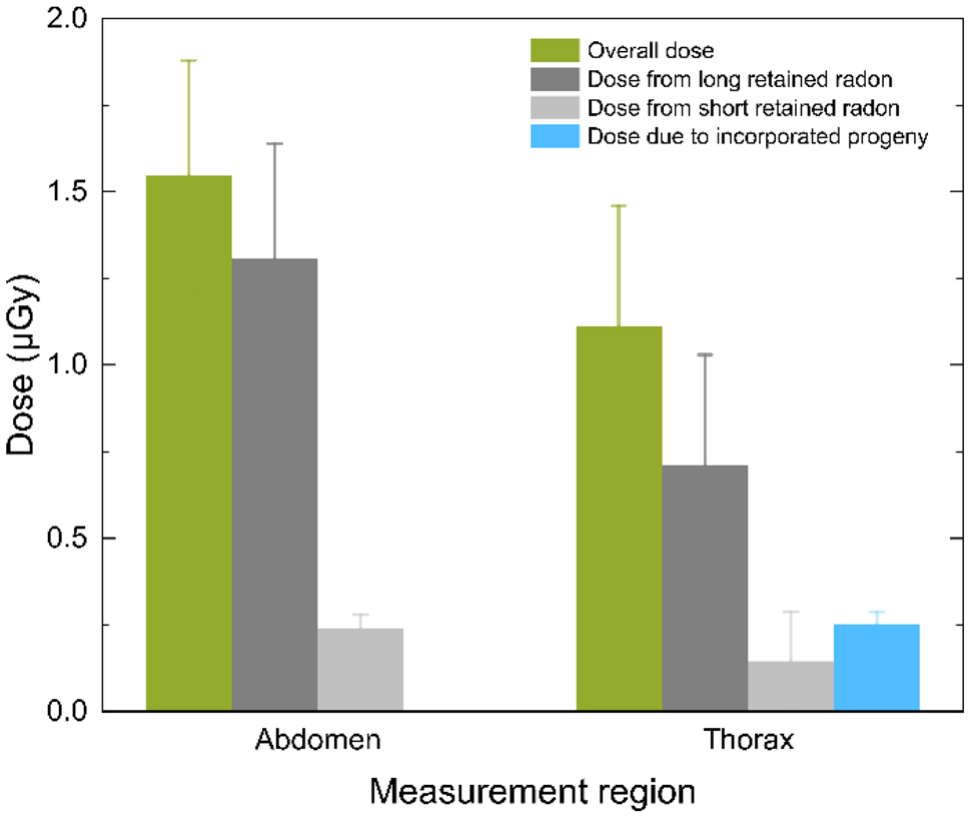
Absorbed dose values for a one-hour exposure at a radon activity concentration of 55 kBq m^−3^ and the assumption of a homogeneous energy distribution in the abdominal and thoracic site. The total absorbed dose is split into the dose due to long (dark grey) and short-retained radon (light grey) and directly incorporated progeny (blue)

Each measured site is considered to consist of two functional compartments with short and long retention times for radon that are responsible for a certain dose fraction. As shown in Fig. 3, the total absorbed dose received in the abdomen for a 1-h exposure at a radon activity concentration of 55 kBq m^−3^ is $1.5 \pm 0.3$ µGy, as mentioned above, whereby $1.3 \pm 0.3$ µGy is assigned to the long-term component and $0.24 \pm 0.04$ µGy to the short-term component. Likewise, in the thorax, the total absorbed dose is $1.1 \pm 0.4$ µGy, where the long-term component of incorporated ^222^Rn is responsible for $0.71 \pm 0.32$ µGy and the short-term component for $0.15 \pm 0.14$ µGy. This shows that over 80% of the absorbed dose from incorporated radon gas is assigned to the compartment with the long retention time. By comparing both measured sites, the indicated higher dose value in the abdomen is due to the stronger long-term component attributed to the higher amount of fat in this body site. Therefore, the measurement at distinct body sites allows us to directly link the observed radon kinetics to their physiological characteristics and to experimentally justify that fatty tissue is a major origin of long-retained radon in the body.

Using the equivalent dose values for the whole abdomen and thorax, the dose conversion factors for incorporated radon gas in both measured sites were determined. However, these factors are restricted to the assumption of a homogeneous energy distribution in the measured site that had to be assumed to calculate the ^214^Pb and ^214^Bi activities from the recorded γ-spectra (see “Methods” section). The influence of a possible inhomogeneity in the energy distribution will be discussed in the following section.

To determine the equivalent doses for both measured sites, the experimentally determined absorbed dose values are multiplied by the radiation weighting factor of 20 for α-particles (ICRP 2003), thereby accounting for the high effectiveness of that radiation type. Then, the dose conversion factor is determined by dividing the calculated equivalent doses of the measured site by the incorporated activity of 22.9 kBq for a one-hour exposure at 55 kBq m^−3^ (see method section where this number is derived). The dose conversion factors for incorporated radon gas in the abdomen and thorax are given in Table 3. A direct comparison of these values with the current predictions from the ICRP is not possible because the ICRP model divides the body into compartments that differ from the measured sites in our experiments. However, by comparing our experimentally obtained dose conversion factors for whole-body sites (abdomen, thorax) with the predicted effective dose for the whole body by the ICRP model that also takes tissue weighting factors into account, the values lie in the same order of magnitude while showing consistently by a factor 2–3 higher values in the investigated individual.

**Table 3:** Experimentally obtained dose conversion factors for incorporated radon gas at distinct body sites under the assumption of a homogeneous energy distribution in the measured site and without using tissue weighting factors and comparison to the ICRP-value (ICRP 2017) for the effective dose (whole-body dose)

	Experimentally obtained values		ICRP
	Abdomen	Thorax	Effective dose (whole body)
Dose conversion factor (SvBq^−1^)	$1.35 \pm 0.29 \cdot 10^{ - 9}$	$7.46 \pm 3.02{ } \cdot 10^{ - 10}$	$4.36 \cdot 10^{ - 10}$

In addition to the dose from incorporated radon gas, there is also a dose contribution from directly inhaled radon progeny in the thoracic site, including the lung. Estimations showed that the dose contribution from inhaled radon gas that decays in the airways is approximately a factor of 15 lower than the dose contribution from directly inhaled progeny in our experimental setup. Therefore, we neglected the dose contribution from decaying radon gas in the airways in our models. In the investigated exposure scenario with a relatively low amount of decay products in the exposure atmosphere (see “Methods” section), directly inhaled progeny accounted for $23.0 \pm 7.8$% of the deposited energy in the thorax and thus $0.25 \pm 0.03$ µGy of the absorbed dose by assuming a homogeneous energy distribution in the measured site (see Fig. 3). However, directly incorporated decay products deposit their energy solely in the respiratory tract and are not homogeneously distributed in the thorax, as will be discussed in the following section.

### The influence of the energy distribution within the measured site

First, the effect of the compartmentalisation of the measured sites on the absorbed doses from incorporated radon gas is investigated. On the basis of our data, the measured sites were divided into two compartments with differing retention times for radon gas, whereby the long-term component can be attributed to poorly perfused fatty tissue. As the subcutaneous fat is primarily located directly under the skin and the compartment correlated with the short-term component is located in the inner body, we have to account for this inhomogeneity. Because the detector efficiency is dependent on the locations relative to the detector cap (see Fig. 1a, b), the long-term component is detected more efficiently and the short-term component less efficiently than predicted under the assumption of a homogeneous energy distribution at the measured site. By correcting for this circumstance, activity values for the long-term component would be at most a factor 2.9 lower than calculated, and activity values for the short-term component would be at most a factor 1.6 higher. However, even under these extreme conditions, the long-term component would still be responsible for more than 50% of the absorbed dose in the measured site instead of 80%, as calculated above.

Additionally, the masses of the functional compartments in which the radon is retained with differing biological half-lives are smaller than the mass of the whole measured site. Based on the mass of the subcutaneous fat from the ICRP reference man (ICRP 1975) and the body fat distribution from Störchle et al. (2018), the here called “regional dose” from long-retained radon in the subcutaneous fat compartment would be 12 times higher and the corresponding regional dose from short-retained radon in the inner body compartment would be slightly higher (about a factor of 1.1) than by assuming a homogeneous dose distribution from short- and long-retained radon over the whole measured site. In combination with the aforementioned efficiency consideration, the regional dose values due to the long-term component would be approximately a factor $\frac{12}{{2.9}} \approx 4.1$ higher in the subcutaneous fat, and dose values due to the short-term component would be approximately a factor $1.1\cdot1.6 \approx 1.8$ higher in the inner body compartment. Although regional doses due to the short- and long-term components locally increase by a factor 2–4, the equivalent dose from short- and long-retained radon in the whole measured site would decrease considering an inhomogeneous energy distribution because regional doses have to be weighted for their contribution to the whole measured site (Fig. 3 and Table 3). Nevertheless, realistic fat distribution scenarios always lead to higher values than those given by the ICRP (ICRP 2017) as we checked by inspecting the impact of compartment geometry on the conversion factors and in a more detailed analysis in the following section. Therefore, it is possible that radon gas contributes more to the effective dose than assumed by conventional models for dose assessments. In combination with the scarce experimental verification of those models, this might show the need for further experimental testing and raises the question of considering the action of primary radon gas in the assessment of the risk related to radon exposure.

However, not only the compartmentalization of the measured sites has an influence on the obtained dose values but also the distribution of directly incorporated progeny in the thorax has to be taken into account. The directly incorporated decay products deposit their energy solely in the respiratory tract and are not homogeneously distributed in the thorax. By assuming exclusive deposition in the lung and applying a lung mass of 0.5 kg (ICRP 1975) instead of the mass for the whole thoracic site of 40.32 kg seen by the detector (see “Methods” section), the calculated absorbed dose value due to directly inhaled progeny is approximately a factor 80 higher and given with $20.2 \pm 2.4$ µGy. The uncertainties for this value are given by the standard error of the fit procedure but do not account for physiological uncertainties, as the value is highly dependent on the breathing rate, which changes with physical activity. This demonstrates the emergence of relatively high absorbed lung doses compared to the surrounding tissue. Considering the radiation weighting factor and the tissue weighting factors of 0.12 for the lung and 0.88 for all other organs and tissues (ICRP 2007), the lung equivalent dose makes up for approximately 2/3 of the effective dose. However, one must bear in mind the special exposure scenario in our experimental setup: Because of the active filtration of progeny out of the exposure atmosphere, the F factor and thus absorbed and equivalent lung doses due to directly incorporated progeny are approximately a factor of 2–4 lower than under standard exposure conditions assumed in the ICRP publications (ICRP 2017). Estimations show that under normal exposure conditions, the lung equivalent dose would be responsible for over 95% of the effective dose, which is in agreement with simulations from the ICRP.

### Comparison with current ICRP model

**Fig. 4:**
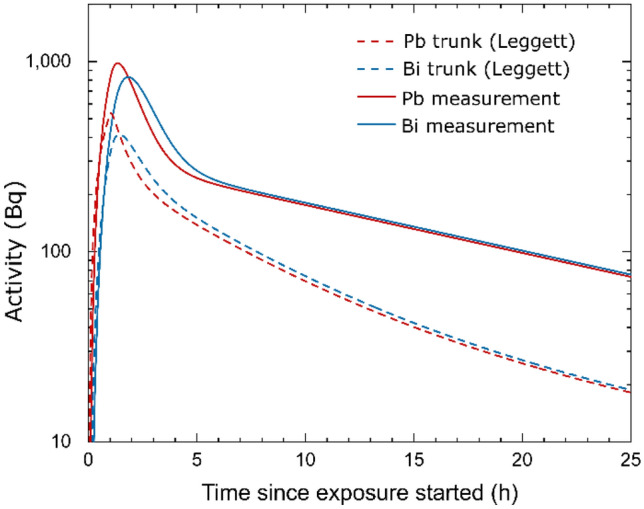
Simulated activity curves for ^214^Pb (red) and ^214^Bi (blue) from the simplified Leggett model adapted to the volunteer’s trunk (dashed line) and our fit of the scaled (55 kBq m^−3^, 40.32 kg) and pooled data for the abdominal measurement (solid line)

To compare our results with the standard biokinetic model in radiation protection of Leggett et al. (Leggett et al. 2013) used by the ICRP (ICRP 2017), the model was simplified and adapted to the abdominal body site of the voluntary patient (see “Methods” section). The simulation for the ^214^Pb and ^214^Bi activity curves in the abdomen for a one-hour inhalation of 55 kBq m^−3^ is compared to the activity curves derived from our abdominal measurements (see Fig. 4).

One difference of the ICRP model compared to our measurements is the existence of compartments with retention times of radon that lie between the detected short-term and long-term components. These retention times were not observed in our measurements, which could be attributed to the fact that signals emitted by compartments that are located in a body area that is far away from or at an unfavourable angle to the detector might not be efficiently detected (see Fig. 1a, b). Seemingly, the slope of the long-term component in the ICRP model is higher than that for our measurements, although the values for the slope of the long-term component in the model and our measurement at the abdominal site show nearly no difference (here: $8.4 \pm 2.3 \cdot 10^{ - 4}$ min^−1^, ICRP: $7.85 \cdot 10^{ - 4}$ min^−1^). Therefore, the seemingly higher slope in the ICRP model is the result of a coexistence of components with different retention times that are not observed in our abdominal measurement. Nevertheless, we observe a higher slope for the long-term component at the thoracic site ($1.3 \pm 0.4 \cdot 10^{ - 3}$ min^−1^) that cannot be correlated with any organ or tissue in the ICRP model. This observation could also be attributed to a mixture of activities from different compartments that could not be separated in our measurement as previously described.

The measured activity values are systematically above the simulated values from the ICRP model. Therefore, the activities of the long-term component are approximately fourfold higher than those given by the ICRP model. This deviation cannot be explained even by regarding the aforementioned efficiency considerations that suggest maximal a factor of 2.9 lower ^214^Pb and ^214^Bi activity values for our measurements of the long-time component. We also obtained at least a factor of 1.5 higher activity values for the short-term component than predicted by the model, which cannot be explained to our knowledge by efficiency considerations or the volunteer’s individual physiology (see below). Therefore and with regard to the investigated individual, we observed a deviation from currently used models to predict the absorbed and equivalent doses from radon gas in different organs and tissues after exposure. This also explains the higher dose conversion factors for radon gas in our measurements compared to those suggested by the ICRP.

Nevertheless, we also have to consider the physiological characteristics of the exposed person and the measured site. The higher fat content in the abdomen than in the thorax seems to be responsible for higher dose values in that body site, as the long-term component, discussed to be attributed to fatty tissue, is responsible for the major dose application. Therefore, the measured site influences experimentally determined activity curves and dose values. That was also a reason to adapt the existing Leggett model to the trunk of the volunteer on the basis of body fat distribution from Störchle et al. (2018). The distribution of the fatty tissue in the body of the volunteer also leads to uncertainties by comparing the activity curves for the trunk of the reference man with the abdominal measurement. An accumulation of fatty tissue at the stomach of the volunteer, i.e., would result in a higher measured long-term component than would be expected by assuming the reference man. But as the weight of the investigated volunteer and the reference man show a deviation of at most 2%, the volume of the body compartments and of the fat compartments should be in good agreement and are not expected to be responsible for the observed deviations. Despite having a chronical inflammatory disease, the volunteer has no disease that would lead to profound physiological deviations between the investigated individual and the reference man. Although we cannot exclude small deviations within the limits of inter-individual variations among persons of comparable body mass, we measure constantly higher activity values in the short- and long-term component than predicted by the simulation. Therefore, it seems unlikely, that the observed deviations originate solely from the individual physiology and anatomy of the volunteer. But as individual physical characteristics may vary between individuals, we cannot generalize our results and transfer them cautiously to other individuals.

## Discussion

The determined retention times for radon in the two compartments of the abdominal and thoracic site can be compared to the retention times obtained from an exhalation measurement of two individuals by Harley et al. (1994). There, the obtained time evolution of radon activity in the exhaled air is a result of the radon transfer from all organs and tissues to the respiratory tract via the venous blood and thus represents a superimposition of all body compartments. They obtained five different retention times for radon in the body given with $23$ s, $4.5$ min, $41$ min, $3.4$ h and $18$ h. In contrast, we observed two retention times for the abdomen and thorax, each that lie in between these values. Moreover, only our measurements allow in-vivo determination of absorbed and equivalent doses at the different measurement sites instead of doses in exhaled accumulating radon emerging from all body parts as we measure the radon decay at specific body sites. Nevertheless, the first two components predicted by Harley and Jetter could technically not be detected using our measurement setup.

By comparing our obtained retention times to the currently used biokinetic model (ICRP 2017; Leggett et al. 2013), the long-term component in the abdomen agrees well with the biological half-life of radon in the poorly perfused fat compartment in the ICRP model that is given with 14.7 h. In contrast, the long-term component in the thorax cannot be assigned to a specific compartment. This might be due to physiological differences of our patient in comparison to the reference man used in the model simulation. Other explanations might be either a superposition of two compartments that could not be resolved in our data, or inhomogeneities within one compartment. The subcutaneous fat compartment, for example, is extended over the whole body so that varying perfusion rates and compositions, resulting in variations in radon solubility, can occur depending on the location in the body.

By looking at an earlier publication, the NRC report 1999 (NRC 1999), determination of transition rates of radon from different body compartments to the blood rely on the same principle as described for the Leggett model. Only the used compartment volumes differ marginally, resulting in minor deviations in the determined transition rates. Another difference is the merging of the two fat compartments that were divided in the Leggett model, so that a biological half-life of 5.4 h is obtained for the fat compartment, which we do not observe in our measurements. Therefore, transition rates determined in the NRC 1999 report do not match our experimentally gained data.

Remarkably, comparing the activity curves and dose values with predictions from currently used dose assessment models (ICRP 2017), we observed at least a factor of 2–3 higher equivalent dose values for the abdomen and thorax of our volunteer than the predicted effective dose for the reference man. Moreover, we measured more short- and long-retained radon gas than the simulations of the activity curves from the ICRP model for the trunk of the reference man suggested. A comparable result was obtained in recently conducted exhalation measurements and whole-body γ-spectroscopy after radon inhalation by Fojtik et al. (2020), which showed lower exhalation rates and more incorporated radon than predicted by the ICRP, although no dosimetry was performed in this experiment. Despite the received dose from radon exposure is dependent on the individual body composition and condition (e.g., low/high fat content, blood circulation rate), we assume that such variations cannot explain the observed deviations. Therefore, the calculated equivalent doses from incorporated radon gas in the measured sites for the investigated individual seem to be higher than currently assumed, leading to the need for a reassessment of the accompanying radiation action. Still, the sparse experimental verification of the radiation protection model justifies our results to question current dose prediction models as also other studies imply higher received equivalent and thus also effective doses after radon exposure (Fojtik et al. 2020).

In radon therapy, a treatment consists of ten one-hour lasting radon exposures at a maximal radon activity concentration of 100 kBq m^−3^. Based on our results presented in Table 3, this would result in a maximal absorbed dose of approximately 20–30 µGy from incorporated radon gas by assuming a homogeneous energy distribution in the measured sites. With a linear energy transfer of 73 keV µm^−1^ for a 6.5 MeV α-particles in water (Berger et al. 2020), a cell nucleus radius of 5 µm and a density of water, approximately every 2000th cell nucleus is hit by an α-particle emitted by radon and its progeny (Schardt et al. 2010), so direct irradiation cannot fully explain the underlying mechanism of radon therapy. Therefore, nontargeted effects (Kadhim et al. 2013) such as the release of immune modulating cytokines after radon therapy may play a role (Rühle et al. 2017; Kullmann et al. 2019).

Nevertheless, we always must bear in mind that the deposited energy in the measured sites is not distributed homogeneously over the whole area. Thus, single organs or tissues with high dose accumulations cannot be resolved using the measurement method presented here. In addition, the data were obtained in only one volunteer under the same exposure conditions. To quantify the effect of intra-and inter-individual differences, the measurements should be repeated with a varying physical activity of the volunteer during exposure and expanded to more individuals.

The data presented here allow us to draw conclusions on temporal and spatial dose distributions after radon exposure in the investigated individual. As the biokinetic models used in radiation protection are verified only by data sets of seven individuals in total that show variations, our explorative measurement gives reason to further test and improve current models in radiation protection and accompanying risk assessment with a higher number of individuals. Knowledge about the radon distribution within the body is a precondition for a thorough mechanistic study of both anti-inflammatory and risk mediating processes and associated biological pathways.
